# Arbor-TVB: a novel multi-scale co-simulation framework with a case study on neural-level seizure generation and whole-brain propagation

**DOI:** 10.3389/fncom.2025.1731161

**Published:** 2026-02-02

**Authors:** Thorsten Hater, Juliette Courson, Han Lu, Sandra Diaz-Pier, Thanos Manos

**Affiliations:** 1 Simulation and Data Lab Neuroscience, Jülich Supercomputing Centre (JSC), Forschungszentrum Jülich GmbH, Jülich, Germany; 2 ETIS Lab, ENSEA, CNRS, UMR8051, CY Cergy-Paris University, Cergy, France; 3 Department of Computer Science, University of Warwick, Coventry, United Kingdom

**Keywords:** Arbor, mouse brain connectome, multi-scale neural models, seizures, The Virtual Brain

## Abstract

Computational neuroscience has traditionally focused on isolated scales, limiting understanding of brain function across multiple levels. While microscopic models capture biophysical details of neurons, macroscopic models describe large-scale network dynamics. Integrating these scales, however, remains a significant challenge. In this study, we present a novel co-simulation framework that bridges these levels by integrating the neural simulator Arbor with The Virtual Brain (TVB) platform. Arbor enables detailed simulations from single-compartment neurons to populations of such cells, while TVB models whole-brain dynamics based on anatomical features and the mean neural activity of a brain region. By linking these simulators for the first time, we provide an example of how to model and investigate the onset of seizures in specific areas and their propagation to the whole brain. This framework employs an MPI intercommunicator for real-time bidirectional interaction, translating between discrete spikes from Arbor and continuous TVB activity. Its fully modular design enables independent model selection for each scale, requiring minimal effort to translate activity across simulators. The novel Arbor-TVB co-simulator allows replacement of TVB nodes with biologically realistic neuron populations, offering insights into seizure propagation and potential intervention strategies. The integration of Arbor and TVB marks a significant advancement in multi-scale modeling, providing a comprehensive computational framework for studying neural disorders and optimizing treatments.

## 1 Introduction

The human brain consists of billions of neurons and an equally vast population of non-neuronal cells, intricately organized into layers and regions (Herculano-Houzel, 2009, 2012). Each neuron operates as a highly sophisticated biochemical machinery (West et al., 2002; Augustine et al., 2003; Darnell, 2013; Lu et al., 2025), coordinating signal transmission within an extensive network in health (Reyes, 2003; Barral et al., 2019; Dicks, 2022) and disease (see e.g., Tetzlaff et al., 2025). Ever since the Hodgkin-Huxley model was introduced to describe membrane potential dynamics (Hodgkin and Huxley, 1952c), computational neuroscience has played a pivotal role in enhancing our understanding of brain function. Yet, due to the immense complexity of the brain and computational constraints, most modeling studies focus on a single scale simulator or rely on standalone simulation codes.

Modeling the large-scale electrical activity of the brain is a complex task. It not only demands familiarity with advanced mathematical methods, but also a solid grasp of the brain's physiology and anatomy. Neural field theory offers a way to study the nonlinear behavior of large groups of neurons at a population level, while still keeping the mathematics manageable. These models give us a strong theoretical framework for understanding key processes in neural tissue, including how the brain transitions between different activity states, such as those seen in sleep or during epileptic events, see e.g., Cook et al. (2022) for a recent review. Moreover, multi-scale computational modeling provides a framework for connecting neural mechanisms with measurements ranging from unit recordings to electroencephalogram (EEG), magnetoencephalography (MEG), and functional magnetic resonance imaging (fMRI). Such models clarify how neural systems compute and interact, and they are essential for integrating empirical findings into a robust theoretical understanding of brain function, see e.g., Deco et al. (2008) and Cooray et al. (2023). Along this direction, recently in Cooray et al. (2025), the authors also studied oscillatory activity in cortical tissue arising from not uniform neural connections. and showed that oscillations can be maintained under a wide range of anisotropic and time-varying connectivity patterns.

Simulations incorporating biophysical properties and neural morphology typically concentrate on individual neurons using simulators such as the NEURON simulator (Carnevale and Hines, 2006). At the microscopic level, studies have explored questions such as how the ion channel kinetics influence neural excitability (see e.g., Gurkiewicz et al., 2011; Suma et al., 2024), how proteins, enzymes, and calcium concentration are distributed among neighboring spines to impact plasticity (see e.g., Luboeinski and Tetzlaff, 2021; Chater et al., 2024), and how signal propagation along axonal fibers relates to neuropathic pain (see e.g., Tigerholm et al., 2014, 2015). Some studies examine how neural morphology—such as dendritic tree growth (see e.g., Yasumatsu et al., 2008) and morphology-dependent plastic interactions (see e.g., Hananeia et al., 2024)—affects function. These studies, while often limited to small patches of the neural membrane, a few dendritic segments, or a small local network, provide valuable approximations of broader neural phenomena.

At the mesoscopic level, researchers simplify neuronal representations using point leaky-integrate-and-fire neurons [based on simulators such as NEST (Gewaltig and Diesmann, 2007) or Brian/Brian2 (Stimberg et al., 2019)], allowing studies on larger networks without explicit neuronal morphology or with some degree of self-customized morphology, using, e.g., NESTML (Linssen et al., 2024). This approach has advanced our understanding of neural heterogeneity (Demirtaş et al., 2019; Nayebi et al., 2021; Gast et al., 2024), self-organization (Zheng et al., 2013; Diaz-Pier et al., 2016; Miner and Triesch, 2016), neural capacity (Emina and Kropff, 2022), energy efficiency (Sacramento et al., 2015), and neural plasticity in disease and health (Manos et al., 2021; Lu et al., 2024). Most microscopic and mesoscopic models remain theory-driven, using mathematical approximations to infer neural behavior rather than directly establishing model based on large datasets (see e.g., Popovych et al., 2019 for a recent review).

Data-driven modeling has gained traction at the macroscopic level with the rise of open-source brain imaging databases [such as OpenfMRI (Poldrack and Gorgolewski, 2017)]. High-resolution structural and functional data from magnetic resonance imaging (MRI) and diffusion tensor imaging (DTI) enable whole-brain modeling based on real anatomical features. The Virtual Brain (TVB) (Sanz Leon et al., 2013; Sanz-Leon et al., 2015; Ritter et al., 2013) and Virtual Brain Twin (VBT) (Hashemi et al., 2025) platforms, for instance, integrate functional MRI and DTI datasets to build individualized models, using coupled oscillators to represent regional activity. TVB has contributed significantly to the understanding of neurological disorders and serves as a testing ground for therapeutic interventions (see e.g., Stefanovski et al., 2021; Monteverdi et al., 2023; Courson et al., 2024 and references therein), for studying self-organization on macroscale (see e.g., Fousek et al., 2024), consciousness (see e.g., Breyton et al., 2024) and healthy aging (see e.g., Lavanga et al., 2023).

With advances in computing resources and simulation technologies, the integration of models across different scales has become both feasible and essential to strike a balance between retaining detailed information and achieving a broad-scale understanding. Recently, a co-simulation framework was introduced that employs NEST and TVB to bridge mesoscopic and macroscopic modeling. This work has pioneered cross-scale modeling (Kusch et al., 2024) and has demonstrated the benefits of integrating models across spatial levels. A notable application of the virtual deep brain stimulation model (Meier et al., 2022; Shaheen et al., 2022; Wang et al., 2025) demonstrated its utility in multiscale simulations. Similar tools have been made available within the European digital neuroscience platform, EBRAINS (Schirner et al., 2022).

However, integrating microscopic and macroscopic models remains technically challenging. At the core lies the vast amount of information being processed, billions of cells with thousands of connections, and the immense gap in timescales, from microseconds in ion channel dynamics to minutes or hours for plastic changes of the connectome. To solve this challenge, we used the Arbor simulator (Abi Akar et al., 2019) and, more specifically, its most recent next-generation version (Cumming et al., 2024), at the microscopic end. Designed for single-neuron and large-scale network simulations, Arbor leverages GPU resources to enhance computational speed and energy efficiency.

In this Methods paper, we successfully established efficient communication between Arbor and the TVB that respects their different operational time scales and provided a use case example of the cross-scale interaction. To demonstrate a first showcase, we used a mouse brain connectome provided by TVB, where each region represents the mean mass neural activity of a brain area modeled by a macroscopic model. Using the co-simulation interface, we replaced one TVB node with a network of detailed neurons modeled in Arbor. An extended Hodgkin-Huxley-based neuron model (Depannemaecker et al., 2022) was utilized in Arbor to simulate different neural activity patterns (e.g., spiking, bursting, seizure-like, etc.). By tweaking a single parameter, we showcased that the seizure-like events generated in Arbor propagated to other nodes modeled in TVB. This platform provides users with the freedom to use existing models across scales with minimal additions and enabled future development in building brain digital twins that contain both micro- and macroscopic information for therapeutic applications.

## 2 Materials and methods

### 2.1 The Arbor simulator

Arbor is an open-source library for building simulations of biophysically detailed neuron models (Abi Akar et al., 2019). It provides an alternative to software like NEURON (Carnevale and Hines, 2006), but with a strong emphasis on modern hardware and scalability to large-scale systems (Hines, 1984). Its overall set of capabilities allows Arbor to model neural networks at a level of resolution beyond point models to explore phenomena like dendritic computation. Thus, support for bulk-synchronous parallelism via MPI, shared memory parallelism by utilizing a thread-pool and job system is central to Arbor, and certain cell types—primarily cable cells—can further leverage SIMD and GPU hardware. Arbor is written in *C*++, though most users interface with it through an intuitive, high-level Python interface built on top of the lower level implementation. The underlying numerical model of Arbor is the cable equation:


$$\begin{array}{l} c\frac{\partial U}{\partial t}=\frac{\partial }{\partial x}\left(\sigma \frac{\partial U}{\partial x}\right)+i \end{array}$$ (1)

where the membrane potential *U* is computed over the morphological structure of the neural tree; the spatial coordinate *x* and the derivative are to be understood within this structure (Von Helmholtz, 1850; Thompson and Kelvin, 1855; Hodgkin et al., 1952; Hodgkin and Huxley, 1952a,b; Loligo, 1952; Hodgkin and Huxley, 1952c; Scott, 1975). The parameters *c* and σ define the membrane capacitance and longitudinal resistance. The trans-membrane current density *i* models the entirety of ionic and non-ionic currents. In both NEURON and Arbor, these are calculated from user-specified sets of differential equations, potentially varying along the morphology. The equations for *i* and *U* are solved in alternation (Lie-Trotter splitting) using a first-order implicit method.

### 2.2 Single neuron and network models in Arbor

**Figure 1:**
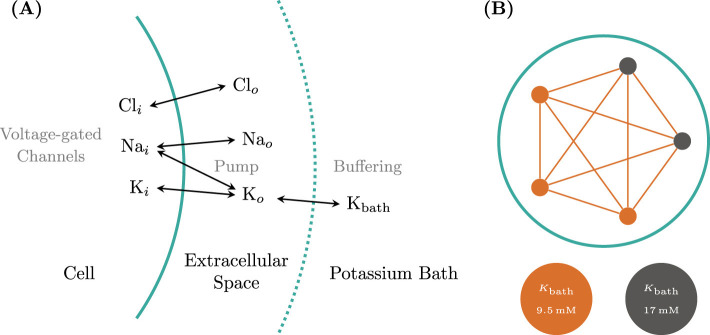
Biophysical neuron model and Arbor network. **(A)** For single cell dynamics, three ion concentrations (K, Na, and Cl) are modeled in the cell's interior and a thin shell of its extracellular medium. The latter is, in turn, surrounded by a bath of a fixed potassium concentration K_bath_. The model simulates changes to the concentration in addition to the current contributions based on three voltage-gated ion channels, an active pump between potassium and sodium, and the buffering effect of the surrounding potassium bath. **(B)** We choose typical values of *K*_bath_ for the single models to generate the tonic spiking and seizure-like event behaviors. In most cases, a fully connected network using exponential synapses with weight *w* = 0.5 is used. As an example, we show here the network instantiation for a network size *N* = 5 and a ratio of SLE to tonic neurons of *f* = 0.2.

**Figure 2:**
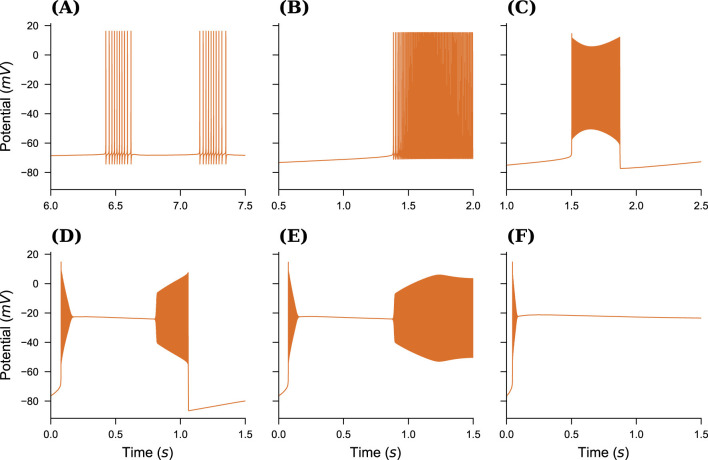
Different neural spiking patterns. **(A)** Spike Train, K_bath_ = 7.5 mM. **(B)** Tonic Spikes, K_bath_ = 9.5mM. **(C)** Bursting, K_bath_ = 12.5mM. **(D)** Seizure-Like Event (SLE), K_bath_ = 17.0mM. **(E)** Sustained Ictal Activity (SIA), K_bath_ = 17.5mM. **(F)** Depolarization Block, K_bath_ = 22.5mM. Note that by setting K_bath_ = 4mM, one can obtain Resting State (RS) activity too (result not shown here).

We begin by selecting a dynamical model that allows for relatively easy yet realistic simulation of a broad spectrum of neural activity at the single-neuron level, governed by a small set of biophysical parameters. The neural model from Depannemaecker et al. (2022) was formulated for Arbor in the Neuron MODeling Language (NMODL). The following equations form the slow part of the system, describing the evolution of ion concentrations due to voltage-gated channels, active pumps, and buffering by an external bath, see Figure 1 for a schematic of the dynamics. It describes the ionic exchanges between the intracellular and extracellular spaces (ICS, ECS) of a neuron immersed within an external bath, acting as a potassium buffer of concentration K_bath_. Ions flow between the ICS and ECS through a sodium-potassium pump and the sodium, potassium and chloride voltage-gated channels, driving changes in the internal (K_*i*_, Na_*i*_, Cl_*i*_) and external (K_*o*_, Na_*o*_, Cl_*o*_) ionic concentrations. By gradually increasing the external bath concentration of potassium ions K_bath_, the model sequentially presents these patterns: resting state (RS), spike train (ST), tonic spiking (TS), bursting, seizure-like events (SLE), sustained ictal activity (SIA) and depolarization block (DB), see Figure 2. The fast dynamics of the membrane potential *V* is modeled in Arbor via the cable equations, see above, which require computing the ion current densities *i*_X_ used in the simulator update as:


$$\begin{array}{l} i_{\text{X}}=g_{\text{X}}\left(V-E_{\text{X}}\right) \end{array}$$ (2)

$$\begin{array}{l} E_{\text{X}}=C\cdot \log\left(\frac{{\text{X}}_{o}}{{\text{X}}_{i}}\right) \end{array}$$ (3)

with the ion species X = {K, Na, Cl} and a non-ion current density:


$$\begin{array}{l} i_{\text{pump}}=\frac{\rho }{\left(1+\exp\left(10.5-0.5{\text{Na}}_{i}\right)\right)\left(1+\exp\left(5.5-{\text{K}}_{o}\right)\right)}. \end{array}$$ (4)

These currents enter the cable equation Equation 1 as the trans-membrane current *i* via:


$$\begin{array}{l} i=i_{\text{pump}}+\underset{X}{\sum }i_{X} \end{array}$$

Following the Hodgkin-Huxley model, conductivities *g*_X_ are written as:


$$\begin{array}{l} \begin{array}{c} g_{\text{K}}=g_{0,\text{K}}n+g_{l,\text{K}} \end{array}\;\begin{array}{c} g_{\text{Na}}=g_{0,\text{Na}}mh+g_{l,\text{Na}} \end{array}\;\begin{array}{c} g_{\text{Cl}}=g_{0,\text{Cl}} \end{array} \end{array}$$ (5)

The—internal *i* and external *o*—ion concentrations are modeled as:


$$\begin{array}{l} \begin{array}{l} {\text{K}}_{i}={\text{K}}_{0,i}+\Delta {\text{K}}_{i}\\ {\text{K}}_{o}={\text{K}}_{0,o}-\beta \Delta {\text{K}}_{i}+{\text{K}}_{g} \end{array}\;\begin{array}{l} {\text{Na}}_{i}={\text{Na}}_{0,i}-\Delta {\text{K}}_{i}\\ {\text{Na}}_{o}={\text{Na}}_{0,o}+\beta \Delta {\text{K}}_{i} \end{array}\;\begin{array}{l} {\text{Cl}}_{i}={\text{Cl}}_{0,i}\\ {\text{Cl}}_{o}={\text{Cl}}_{0,o} \end{array} \end{array}$$ (6)

The variables {ΔK_*i*_, K_*g*_} evolve as:


$$\begin{array}{l} \frac{d\Delta {\text{K}}_{i}}{dt}=-\gamma \left(i_{\text{K}}-i_{\text{pump}}\right) \end{array}$$ (7)

$$\begin{array}{l} \frac{d{\text{K}}_{g}}{dt}=\epsilon \;\left({\text{K}}_{\text{bath}}-{\text{K}}_{o}\right) \end{array}$$ (8)

where γ converts the current density *i*_X_ to molar flux, summarizing the effect of the ion pump in Figure 1A and the external buffer. Finally, fast dynamics were reduced and adjusted to mammalian neurons:


$$\begin{array}{l} \frac{dn}{dt}=\frac{1}{\tau }\left(n-n_{\infty }\left(V\right)\right) \end{array}$$ (9)

$$\begin{array}{l} n_{\infty }\left(V\right)=\frac{1}{1+\exp\left(-\left(19+V\right)/18\right)} \end{array}$$ (10)

$$\begin{array}{l} m=m_{\infty }\left(V\right)=\frac{1}{1+\exp\left(-\left(2+V/12\right)\right)} \end{array}$$ (11)

$$\begin{array}{l} h=h\left(n\right)=1.1-\frac{1}{1+\exp\left(3.2-0.8n\right)} \end{array}$$ (12)

based on the observations that the reaction of the sodium gating variable to changes in *V* is nigh instantaneous and *h*(*t*) + *n*(*t*) = const.

The resulting ion channel was added to a basic, spherical, single-compartment neuron. After implementing this biophysical model, we reproduced the firing patterns using the parameters of the reported model (Figures 2A–F), see also Depannemaecker et al. (2022) for more details and motivation for model parameter choices. Note that despite the values given in the original publication, neither the Arbor nor the published reference implementation produces the depolarization block pattern at *K*_bath_ = 20mM but only at around *K*_bath_ = 22.5mM. From here, a simple model network was developed, comprising *N* total cells, with a mixture of tonic *f* · *N* and SLE (1 − *f*) · *N* cells, where both sub-populations are assigned individual values for *K*_bath_, sketched in Figure 1. Cells are connected using exponential synapses with an internal weight of *w* = 0.5 chosen to produce an activity similar to Rabuffo et al. (2025) which uses delta synapses.

**Figure 3:**
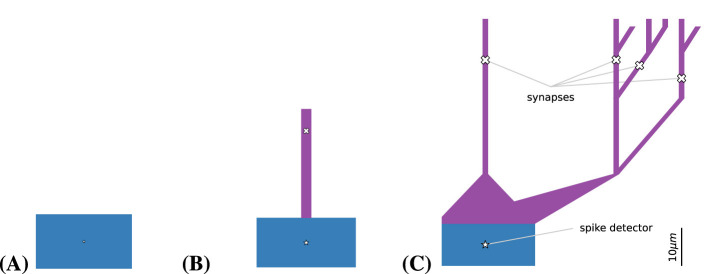
Compartmental neuronal morphologies used for simulations. Each morphology comprises a soma (blue) and a dendritic section (violet). For numerical simulations, dendritic segments are discretized into 5μm compartments, whereas the soma is modeled as a single compartment. Since the cable model neglects extracellular effects, a 1.5-dimensional morphology is employed internally, rendering spatial relationships irrelevant to the model dynamics. When multiple connections converge onto a single cell, synaptic assignments follow a round-robin scheme. **(A)** Soma only. Equivalent to a point model, the cylindrical segment has a radius *r* and a length of 2*r*, chosen to yield the same surface area as a sphere of radius *r*. Both synapses and spike detectors are attached at the center of the soma. **(B)** Ball and stick. A straight dendritic segment is added, featuring passive current flow and a synapse attached at a fixed distance from the soma. **(C)** Random trees. More complex morphologies are generated as random binary trees of depth five. Synapses are placed at a fixed distance from the soma and may be targeted by connections originating from spike detectors, which are positioned at the soma center. See Supplementary Figure S1 for more examples of randomly generated morphologies.

In addition to the elementary spherical morphology, we also investigated two multi-compartmental neuron models. The first model included a single dendritic segment of 25μm, subdivided into 5μm compartments. The second model extended the dendrite into a randomly generated tree composed of 5μm compartments. To introduce variability across cells, the random number generator was seeded with each cell's unique identifier (see Figure 3 for examples). In future work, these synthetic morphologies will be replaced with reconstructions derived from neural imaging data available in public databases. Supporting such models in Arbor will require only a simple command to load per-cell morphology data from disk.

### 2.3 TVB network model

Following Deco et al. (2013), we used the reduced Wong-Wang model (Wang, 2002) to simulate resting-state activity and to investigate the dynamics of local brain regions embedded within a large-scale brain network. The mean firing rate *H*(*x*_*I*_) and mean synaptic gating variable *S*_*I*_ of region *I* are described by:


$$\begin{array}{l} \frac{dS_{I}}{dt}=-\frac{S_{I}}{\tau_{s}}+\left(1-S_{I}\right)\gamma H\left(x_{I}\right) \end{array}$$ (13)

$$\begin{array}{l} H\left(x_{I}\right)=\frac{ax_{I}-b}{1-\text{exp}\left(-d\left(ax_{I}-b\right)\right)} \end{array}$$ (14)

$$\begin{array}{l} x_{I}=\omega J_{N}S_{I}+GJ_{N}\underset{K}{\sum }c_{IK}S_{K}+I_{0}, \end{array}$$ (15)

where *x*_*I*_ is the synaptic input to the *I*-th region, ω = 1 denotes the local excitatory recurrence, *c*_*IK*_ is the strength of the structural connection from the local area *I* to *K*, and *G* is a global coupling strength. The parameters are set to the same values as those used in the TVB implementation. *J*_*N*_ = 0.2609nA is the synaptic coupling of NMDA receptors and *I*_0_ = 0.33nA is the baseline external input. The kinetic parameters are τ_*s*_ = 100ms and γ = 0.641. The parameters of the input-output function *H* are *a* = 0.27nC^−1^, *b* = 0.108kHz, and *d* = 154ms. Depending on the tuning of *G*, the system exhibits a multi-stable regime, with steady states of high and low spiking activity. Here, we set *G* = 0.096.

### 2.4 Co-simulation framework of Arbor and TVB

**Figure 4:**
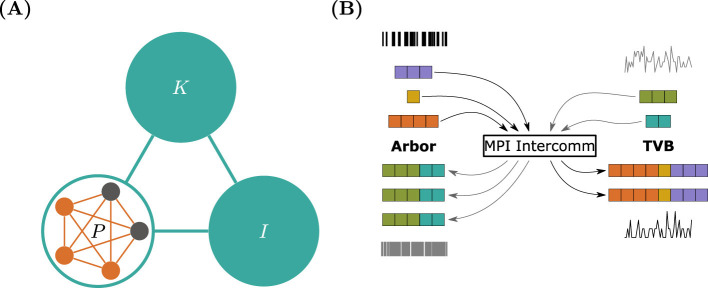
Arbor-TVB co-simulation schematic and communication pattern. **(A)** In a TVB simulation of regions *I*, *K*, and *P*, one region *P* will be replaced by a proxy containing a network of detailed cells simulated in Arbor. Regions are connected via the weights of the connectome and produce an activity values based on the chosen region model. When crossing the boundary between TVB and Arbor models, care needs to be taken to convert between discrete action potentials in Arbor to continuous, region-model-specific variables in TVB. **(B)** Spikes generated by Arbor and TVB—converted from activity values interpreted as mean spiking rates—are exchanged using an MPI intercommunicator and the All-gather primitive. This is equivalent to concatenating all contributions from all Arbor MPI ranks and sending the result to all TVB ranks and *vice-versa*.

Both Arbor and TVB offer support for attaching a second simulator to perform co-simulation, potentially at different scales. Co-simulation from TVB's viewpoint is the simpler technology of the two frameworks, since TVB is designed to execute as a single process. TVB allows for exchange of any variable relevant to the region models and any number of variables. The co-simulation partner is encapsulated in one or more TVB regions, called proxy nodes, see Figure 4. These proxies present a conforming interface to TVB, exchanging the salient variables as a table, one row per time-step, one column per variable. As TVB advances in lockstep on a global time-step, this is almost identical to normal operation. However, co-simulation introduces the concept of an ‘*epoch*’ to TVB, i.e., the length of time that conforms to the smallest delay τ_min_ in the set of inter-region connections delays τ_*IJ*_, with *I* and *J* referring to two connected regions. These delays are part of the connectome data used to construct a TVB simulation. In the case that a connectome contains zero-valued delays these must be replaced with a pre-defined finite value. Further, it is required that the time-step evenly divides τ_min_. Co-simulation thus can integrate all nodes' state, including the proxy, for one epoch τ_min_ without exchanging data. This is correct as an event emanating from any region *I* at time *t* influences any other region *J* at time *t* + τ_*IJ*_ ≥ *t* + τ_min_. Only after an epoch, data need to be exchanged between the proxy and the rest of the TVB regions. A TVB–NEST demonstration has been published to showcase the interaction between a local network of spiking neurons and the whole-brain network dynamics (Kusch et al., 2024).

Arbor has a different design in terms of connectivity. Interaction between physically separate cells is mediated by action potentials, e.g., when the membrane potential triggered by dedicated sources crosses a configurable threshold. Cells are connected by wiring these sources to corresponding sinks like synapses via an abstract connection object comprising a delay and weight, modeling transmission and attenuation via an axon. In contrast to TVB, Arbor is fundamentally a distributed system and internally employs the same approach to decoupling via the minimum network delay as explained above. To initiate co-simulation, Arbor utilizes an additional interface to manage the spike exchange from external connections that are originated from outside but terminate at cells simulated in Arbor. On the technical side, the latter part leverages MPI_Allgatherv through an inter-communicator and effects that the concatenation of all spikes sent from all MPI ranks running TVB arrive on all ranks running Arbor and *vice versa* (Figure 4B). This allows co-simulation in conjunction with arbitrary numbers of ranks on both sides and even in compounds with more than two simulators.

Finally, bi-directional translation between TVB's variable concept and Arbor's representation of action potentials is required. As the former depends on the region models used, we chose to bundle this with the remaining TVB functionality as part of the Arbor proxy node. For the TVB models used in this study, the main variable is the per-region mean activity rate ν_*I*_ which is conceptually compatible with the concept of spike generation. For each region *I* connected to the proxy node *P*, i.e., with connectome weight *c*_*IP*_ > 0, a set of synthetic events must be generated such that the mean activity conforms to ν_*I*_. This is an ambiguous process, even if we prescribe a population (list of cell identifiers) and a per-cell distribution, e.g., a Poisson point process, from which to draw events, which likely must be resolved by ensembles of simulations. In general, this is both model dependent and mathematically intractable, so we leave the general case as a customization point in the framework. For our running example, however, we make the following choice: Event timings for the current step *k* will be drawn from a uniform distribution and dispatched to all cells in the Arbor network. Note that while these events are created at given time a per-connection delay is applied and thus delivery occurs at a later time.

**Figure 5:**
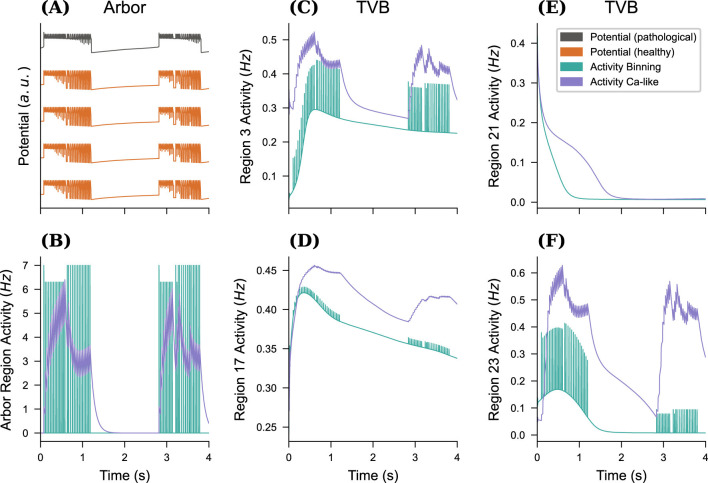
Impact of conversion method on activity exchange. For an all-to-all connected network of a mixture of 10 SLE cells (*K*_bath_ = 17.5mM) and 90 tonic (*K*_bath_ = 9.5mM) neurons in Arbor, we plot the membrane potential traces for four tonic and one SLE cell in **(A)**. This simulation is repeated for two activity exchanging methods, either spikes were binned into buckets of width Δ*t* to extract instantaneous rates, or the differential equation Equation 16 emulating the change in Calcium concentration of a biological cell after spiking was used (with τ = 100ms and $\beta =\frac{0.1}{N}$). The resulting activity traces for the Arbor network **(B)** and selected TVB nodes (out of 98 regions) are displayed in **(C–F)**.

The inverse direction, converting spike events to mean rates, while being well-defined, is still subject to customization. We explore two options here. First, simple running averages, i.e., all spikes that originate within the Arbor network during the current epoch, are collected and sorted into bins of width Δ*t*. This list is then normalized to the cell count and time step and sent to TVB as the mean activities as a function of time. Although straightforward, this can lead to unrealistically rough activity traces, especially if cell populations are small. Second, as inspired by high-speed calcium imaging experiments (Grewe et al., 2010), a mechanism to track cell activity through calcium level is implemented as:


$$\begin{array}{l} \frac{dC_{p}}{dt}=-\frac{C_{p}\left(t\right)}{\tau }+\beta \underset{t_{\text{spike},p}}{\sum }\delta \left(t-t_{\text{spike},p}\right),\;C_{p}\left(0\right)=0 \end{array}$$ (16)

with per cell *p* having a decay parameter τ and a weight β. Computing the activity becomes the average:


$$\begin{array}{l} \nu_{P}\left(t\right)={\langle C_{p}\left(t\right)\rangle }_{p\in P}, \end{array}$$ (17)

yielding a smooth trace. This method of converting discrete spiking events into a continuous interval variable is also used in a few plasticity models recruiting a negative feedback control mechanism such as synaptic scaling (Van Rossum et al., 2000) and homeostatic structural plasticity (Butz and Van Ooyen, 2013; Diaz-Pier et al., 2016; Lu et al., 2024) models. The choice of the calcium kernel parameters is based on trial-and-error to match the output of Arbor and the magnitude of activity generated by TVB. Figure 5 compares the impact of this choice on the macro-scale network. In small networks and over short timescales defined by the epoch length as shown in the example, spiking activity occurs in noncontinuous bursts, which is dubious in conjunction with the smooth dynamics of the chosen TVB model. Figure 5A shows the propagation of this noncontinuous activity into the TVB regions, while using the Ca-like model (B) provides smooth dynamics in both the Arbor and TVB models. We thus will use the latter in all simulations from here on out. In general, both methods require scaling by the number of cells in the proxy region to arrive at a scale-free activity measure. A local scaling factor *G*_A_ is used to convert between the activity of the detailed network and the activity of the region modeled in TVB. In general, *G*_A_ needs to be adjusted to the choice of connectome and TVB model, similar to the choice of the global coupling strength *G* in the RWW model. In this study, *G*_A_ = 100 is used as it produces seizure-like propagation patterns comparable to those found in similar studies, see e.g., Melozzi et al. (2017) and Courson et al. (2024) and references therein.

## 3 Results

So far, we have described the components of the co-simulation framework. It consists of the following components: a TVB model based on the connectome and node dynamics, a specified set of TVB nodes where the Arbor models will be placed, one or more internally connected network models in Arbor, a defined mechanism for routing events from TVB to individual cells in Arbor, a method for translating Arbor-generated events into TVB variables, and a translation process for converting TVB variables into events originating from synthetic cells. Each of these components serves as a customization point for the user. While reasonable default configurations can be provided for some, others require user-defined specifications to suit specific modeling needs.

The single cell model has been demonstrated to exhibit the necessary range of behaviors. We have also motivated our choice for converting spikes to rates of using a biologically-inspired exponential smoothing filter via a Ca-like activity over simple binning and fix parameters to τ = 100ms and $\beta =\frac{0.1}{N}$. This normalization is important, as it produces results that are invariant under changes in the number of cells *N* in the detailed network.

To induce seizure propagation in mice brain, we use the structural connectivity derived from the Allen mouse brain atlas (Oh et al., 2014), also used in Melozzi et al. (2017), to embed the TVB nodes. Following the work presented in Courson et al. (2024), the proxy node modeling the Arbor population of tonic-spiking and SLE point neurons is set within left the Hippocampus and in particular in the left-field CA1 (l CA1), a region that is prone to generate widespread seizures.

### 3.1 Seizure induction among morphologically detailed cells

**Figure 6:**
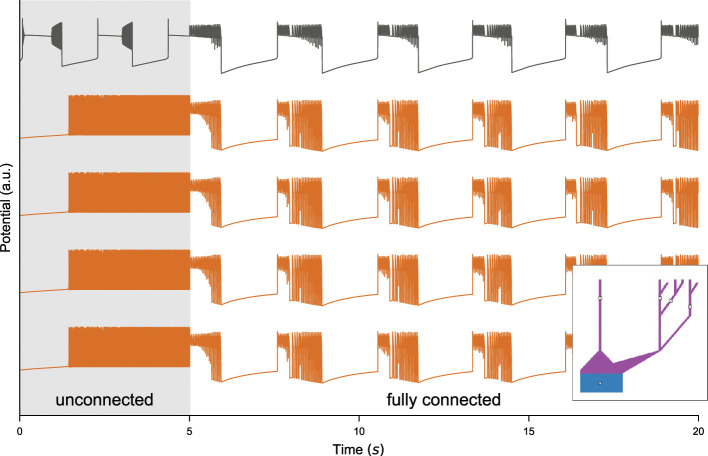
Network level effects induced by SLE activity. Simulation of a 100-cell network with 80 tonic-spiking (*K*_bath_ = 9.5mM) and 20 SLE (*K*_bath_ = 17.0mM) neurons. Membrane potentials are shown for one SLE (top) and four tonic-spiking cells. Each neuron includes a single-compartment soma and a random dendritic tree. The model was integrated for 5s (shaded region) without internal connections, then switched to a fully connected network, which settled into a new equilibrium dominated by SLE activity. Inset: Example morphology of the first cell.

As an initial step, we examined a network of morphologically detailed cells (comprising of a single-compartment soma and a random dendritic tree, see bottom-right inset panel of Figure 6) without embedding them into a co-simulation framework. The objective was to assess how interactions among neurons exhibiting distinct firing patterns influence network dynamics within a small population of SLE neurons at the local (Arbor) level. We constructed a network comprising 80 tonic-spiking neurons (*K*_bath_ = 9.5mM) and 20 SLE neurons (*K*_bath_ = 17.0mM), initially without internal synaptic connectivity. The system was simulated for *T* = 5*s*, and the resulting membrane potentials are presented in Figure 6 (this initial phase is indicated by the gray-shaded region). During this initial phase, all neurons independently showed their intrinsic firing behavior, consistent with the activity shown in Figure 6. Following this baseline simulation, integration was paused, the network was reconfigured to full connectivity, and the simulation was resumed. The introduction of connectivity produced an immediate response across the network. The SLE neurons progressively transitioned toward tonic-like spiking, mirroring the dominant (80%) tonic-spiking subpopulation. At the same time, tonic-spiking neurons began to exhibit the bursting activity characteristic of the SLE phenotype (Figure 6, *T* ≥ 5s)). Note that the use of different *K*_bath_ values for neurons within the same group is, at this stage, driven by computational considerations and the need to validate the performance of the Arbor-TVB co-simulator. This choice can be adjusted to study more biologically realistic scenarios which involve the interaction of different neural populations or transition of network dynamics, which is further addressed in the Discussion section.

### 3.2 Seizure induction and propagation employing point neurons in Arbor-TVB co-simulator

**Figure 7:**
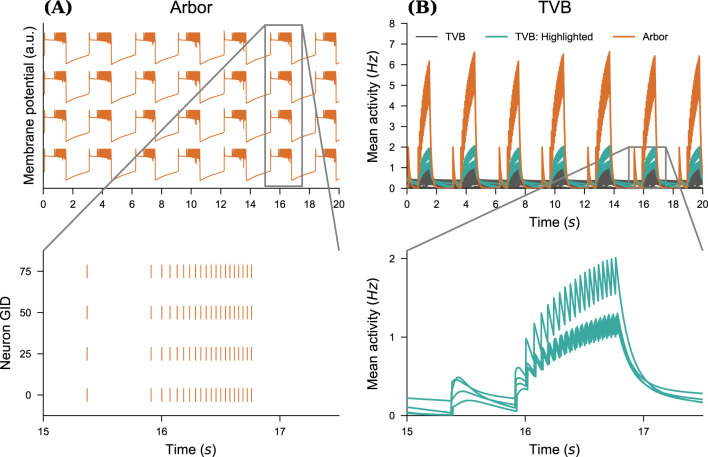
Multiscale seizure propagation. **(A)** Membrane potential and raster plot for four neurons in the detailed fully-connected Arbor neural network. Note that the membrane potential time-series are similar across all neurons. **(B)** Mean firing rate of various TVB local brain areas vs. the Arbor activity. We track a seizure after a transient period, so that all brain areas have reached their baseline activity. The zoomed-in sections show the propagation of the seizure. We highlight and show in detail the four time series with the largest deviation in activity.

Next, we embedded a network of detailed neurons as a proxy node in a simulation of neural mass models in TVB. The general setup is similar as before, however, the proxy node now consists of only SLE-type point neurons in a fully connected network. Figure 7A illustrates the evolution of the membrane potential of individual neurons within the Arbor network, with all neurons exhibiting identical dynamical behavior. The pattern of SLE activity is modulated by neuronal coupling.

We run simulations over 20s, and investigate the effect of SLE emergence in the network once all brain areas have reached their steady state. The production of these patterns in the Arbor node generates changes in the firing rates of local TVB brain areas. Even though changes in activity occur in most brain areas, these fluctuations occur within different ranges.

In Figure 7B, we show the time-series of TVB nodes' firing rate throughout the simulation, here with *N* = 100 in the Arbor detailed network. A transient period is necessary before all nodes reach their steady-state. The emergence of recurrent SLE patterns in the Arbor node triggers a mixture of periodic and seizure-like patterns in the dynamics of the TVB nodes. We highlight traces corresponding to the l CA1 region modeled using detailed cells (orange) and the four regions with the highest activity modeled by the neural mass model. The inset shows the highlighted traces, excluding l CA1, during a single period.

**Figure 8:**
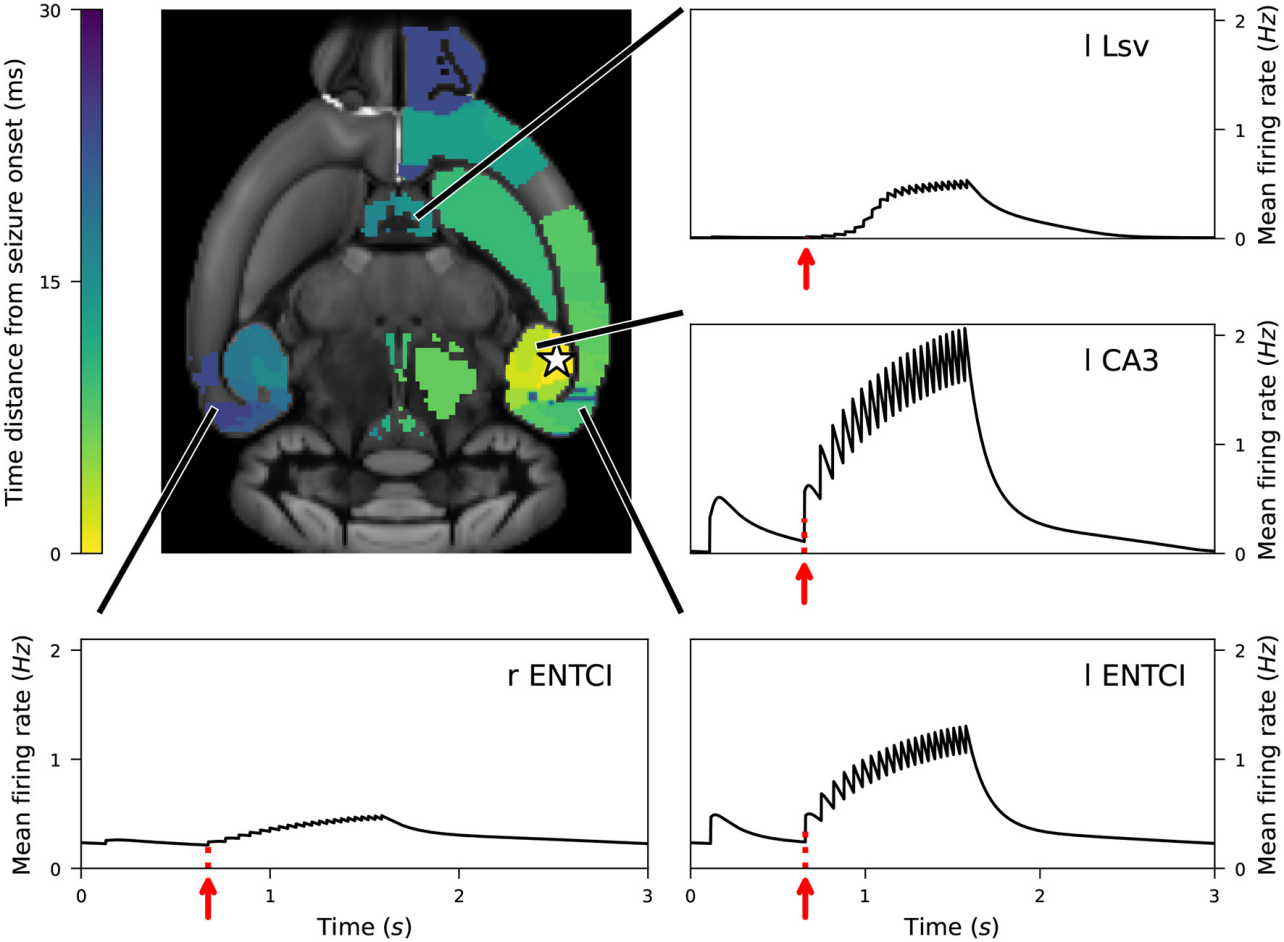
Propagation of a seizure originating in left-field l CA1 area of the mouse brain model. Time distance between seizure emergence in l CA1 (star marker) and spiking rate increase in each brain area. We also depict the firing activity time-series of four initially non-SLE nodes of the brain network being recruited in the seizure, namely the ventral part of left Lateral Septal Nucleus (l LSv), l CA3, l ENTCl and r ENTCl. The red arrows indicate the beginning of the bursting activity. Non-colored regions (panel with the mouse brain template) correspond to areas where seizures either did not occur or had relatively weak effects. See text for more information regarding the onset detection of seizures in other regions. See text for more details.

In Figure 8, we present the propagation of SLE originating in l CA1 (star marker), represented as a fully connected network of *N* = 10000 SLE point neurons. Colors on the brain template show the time distance between seizure emergence in the diseased area and seizure arrival in the different brain regions. The systematic detection of SLE onset in other regions of the whole-brain network (if and when present within the simulation period) is performed as follows. First, the baseline firing activity of each region is defined (first time window of the simulation). The non-SLE nodes initially exhibit a relatively stable firing rate, which gradually becomes influenced by the SLE originating from the Arbor node. When connected to the Arbor node, spiking-tonic brain areas are repeatedly recruited into SLE activity patterns, followed by periods of relaxation. For each region, we define the baseline firing rate as the minimum firing rate observed during the relaxation phase immediately preceding any event in the onset region. When a robust increase in firing rate is detected in a region—indicative of a seizure-like event—the corresponding onset timestamp is recorded. The baseline firing rate serves as a reference, and seizure activity is identified based on a sustained increase in firing rate over a defined duration. We define the onset of a seizure as the beginning of the high-amplitude bursting regime, specifically the point at which the firing rate begins to rise consistently after the first small activity peak. To ensure that only significant deviations are classified as seizures, we require the firing rate to exceed the baseline by at least 10% and remain above this threshold for a minimum of 100 ms. Our seizure onset detection follows a similar (though not identical) approach to that used in related studies—for example, Melozzi et al. (2017) and Courson et al. (2024)—where seizure-like or bursting activity in mouse brain models is identified by monitoring a model variable and applying a threshold to detect the onset. In Figure 8, we also depict the activity time-series of four initially non-SLE nodes of the brain network being recruited in the seizure, namely the ventral part of left Lateral Septal Nucleus (l LSv), l CA3, l ENTCl and r ENTCl. In the main panel (mouse brain template), non-colored regions correspond to areas where seizures either did not occur or had relatively weak effects.

The Arbor network was initialized with fixed connection weights (*w* = 0.5). To assess robustness, we also tested weights drawn from a normal distribution (μ = 0.5, σ = 0.5), truncated to positive values. Neural activity was averaged over 20 independent realizations (see Supplementary Figures S2, S3). Experiments with inhibitory/excitatory network variants are also currently in progress. Both baseline and seizure-evoked activity differ across brain regions. Due to the symmetrical inter-hemispheric connections in the Allen Mouse Brain SC, l ENTCl and r ENTCl share the same baseline firing rate. As the SLE pattern emerges in the left hippocampus, l ENTCl exhibits higher spiking rates.

### 3.3 Computational performance of the Arbor-TVB co-simulation framework

We next evaluated the performance of the running example on a single Apple M1 (2021) laptop. Arbor was built with MPI and SIMD (Arm Neon/SVE) support, with cells organized into groups of ten to fully exploit SIMD capabilities. The overall runtime consists of four primary components: (1) Arbor model update, (2) Conversion from spikes to rates, (3) TVB model update, and (4) Conversion from rates to spikes.

**Figure 9:**
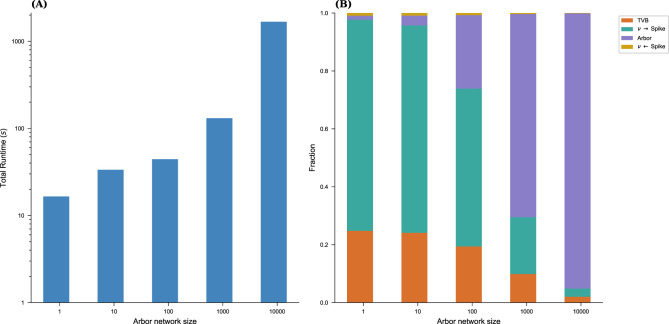
Performance of the running example. We graph the overall runtime **(A)** of the full co-simulation over the number of cells in the Arbor network as well as the fraction of the main contributions **(B)**: time spent in both simulation engines and in converting between rates and spikes. TVB and rate to spike conversion are the most relevant cost center in the limit of vanishing Arbor network sizes while at large sizes, Arbor compute time dominates.

The Arbor update runs in parallel with the conversions between rates and spikes, as well as the TVB update. During spike exchange, both simulations synchronize, meaning the slower part must wait in the MPI collective, which accounts for the primary time spent in the collective call. Figure 9 illustrates the total runtime of a 10s simulation for the entire model described above, along with the relative contributions, for system sizes ranging from one to 10,000 cells. Notably, at 10,000 cells, nearly all computational time is spent within the Arbor network model. In future experiments, we plan to leverage additional hardware, including GPUs, to accelerate the Arbor side of the simulation. At this scale, TVB and the spike/rate conversions are potential bottlenecks that will require optimization, potentially through TVB's JIT compilation and GPU acceleration. Additionally, further parallelization and porting of the conversion steps to a more performant programming environment remain promising avenues for improvement.

## 4 Discussion

In this work, we presented a co-simulation framework that offers a novel approach to bridging the gap between microscopic (spiking neuron) and macroscopic (mean-field) models. This framework integrates simulators Arbor and TVB within a parallelized MPI environment, enabling a detailed yet computationally feasible representation of neural dynamics across scales. To model large-scale brain network dynamics, we used the mean-field reduced Wong-Wang model to reproduce resting-state dynamics. Simultaneously, a detailed spiking neural network was simulated with Arbor, employing a physiological model of seizures at the neuron level (Figure 2). The spiking activity of the population was then converted into a smooth trace for the proxy node, which was communicated with TVB (Figure 4B). This co-simulation approach successfully captured the interplay between spiking activity and large-scale brain dynamics, where local neuron dynamics generate global activity wave-fronts. At the microscopic scale, we demonstrated that the structure of the detailed neural network influences its activity patterns, thereby affecting the shape of the activity wavefront (Figure 5).

As a proof of concept, and as a technical showcase, we simulated the emergence of seizure-like activity patterns (SLE) in the mouse hippocampus, using the Allen Mouse Brain Structural Connectivity data. By tuning the single neural parameter, we modeled the target brain area with a small, fully-connected network of (point and detailed-compartmental) neurons SLE, which produces scale-free activity patterns. This network design can be further adapted to investigate more bio-inspired scenarios, such as the study of: (i) ensembles of neurons within a given brain area that belong to different sub-regions (i.e., are relatively distant from one another), whose dynamical activity may differ significantly from that of other sub-groups and (ii) dynamical transition phenomena where the *K*_bath_ value is set near a critical threshold—e.g., inducing a transition from regular spiking to bursting activity, see Figures 2A, B. The latter consideration is more general, in the sense that for a different dynamical model of neural activity, one can choose a relevant control parameter that drives transitions in neural activity patterns—for example, from slow spiking (representing healthy activity) to fast bursting (associated with pathological activity) in a given region, when the system operates near a critical transition point (e.g., from spike trains to bursting). Our approach offers a well-understood and easily controlled platform by showcasing its technical usability. By adapting the Arbor nodes with different cell composition and connectivity, we demonstrated its significant potential for more complex cell models untapped.

Although using seizure propagation as a use case, the seizure activity patterns established in the present study may not capture precisely the complex nature of all epileptic seizures. For example, there are other types of seizures that occur in different brain disorders, i.e., acute symptomatic seizures, which are not like those caused in epilepsy. For example, patients with Alzheimer's disease may also experience seizures, which are classified as progressive symptomatic seizures and typically arise from underlying neurodegenerative processes., see e.g., (Mauritz et al., 2022) for a recent relevant review.

The implementation in the present study extended the functionality and application scenarios of Arbor. Arbor has embarked on many types of computational studies as a new-generation simulator. It enables seamless conversion and simulation of single-neuron models from the NEURON simulator and supports simulations of both individual neurons and large-scale networks. Arbor accommodates various plasticity models, including spike-timing-dependent plasticity, calcium-based synaptic tagging and capture, and structural plasticity. It has been used to study synaptic tagging and capture via the built-in diffusion functionality (Luboeinski et al., 2024, under review). Recent developments focus on co-simulation with membrane dynamics and external kernels, enabling dynamic connectivity modifications in a distance-dependent manner. With its high flexibility and scalability, Arbor stands out as a promising platform developed within the EBRAINS initiative to advance cross-scale simulations in computational neuroscience. Arbor is available as part of the EBRAINS software distribution (ESD) on connected HPC centers and the EBRAINS collab via Jupyter Lab. Providing a bridge between morphologically detailed neurons and neural mass models encompassing the full brain spans a gap of scale from sub-micrometer to decimeter. It allows for placing the resolution—using Arbor and detailed models—where needed and using realistic, data driven environments everywhere else via TVB.

Despite its successes, the Arbor-TVB framework has some limitations. The co-simulation requires careful exploration and calibration of coupling parameters to ensure meaningful interactions between Arbor and TVB, which remains a challenge when generalizing to diverse neural models. The TVB network we used here is homogeneous, in the sense that all model parameters are set to be identical. While this is not a highly realistic assumption—particularly when assigning a dynamical mean field model to simulate the activity of a brain region—it is a common choice among researchers when modeling whole-brain resting-state dynamics, see e.g., Popovych et al. (2021) and Manos et al. (2023) and references therein. Note that even with identical initial settings, firing rates vary across regions due to the influence of long-distance connectivity weight values and the resulting complex dynamics. Our use-case simulation of seizure propagation is not yet directly compared to experimental data from mice or humans. Nevertheless, the current Arbor-TVB implementation is capable of indirectly capturing biologically realistic brain dynamics and activity propagation, as reported for example in Melozzi et al. (2017) and Courson et al. (2024). The same applies to the choice of calcium kernel parameters. This kernel is inspired by experimental observations via calcium imaging experiments (Grewe et al., 2010) but no exact values are available. Due care should be taken when matching the magnitudes of both Arbor output and TVB output. Moreover, within the Arbor-TVB framework, the chosen dynamical model can be further tuned to generate neural activity—such as BOLD signals or firing rates—that more closely aligns with neuroimaging data, thereby enabling the simulation of more realistic brain dynamics. Specifically, computational costs for large neural networks may necessitate further optimization in model parallelization and data handling. A near-term goal would be to incorporate new features, such as synaptic plasticity, which could offer valuable insights into how brain networks adapt and reorganize in response to disrupted activity.

As stated in the Introduction section, it is natural to compare the Arbor-TVB co-simulation framework presented in the current Method paper with the established NEST-TVB co-simulation (Kusch et al., 2024). Both simulators have their own merits and application scope. NEST has a longer history (Gewaltig and Diesmann, 2007) and a rich profile of neural models and plasticity models. Its performance is excellent in mesoscopic modeling of spiking neural networks. Arbor is younger but has made it possible to efficiently simulate networks of the highly expensive biophysical HH model with multi-compartments (Abi Akar et al., 2019). It also accommodates a variety of plasticity rules, including heterosynaptic dendritic plasticity rules inside the dendritic shaft (Luboeinski et al., 2024). The Arbor-TVB co-simulation framework has thereby inherited those merits of Arbor, while the NEST-TVB co-simulation framework opens the venue for users to freely use NEST functions for co-simulation. Those two tools are complementary for distinct research purposes. Moreover, since Arbor is designed to make the best use of both GPU and CPU, Arbor-TVB can also be easily adapted to make use of the cutting-edge exascale GPU computing resources.

From an epilepsy-seizure perspective, while the framework provides insights into seizure propagation, additional validation against empirical data would enhance its applicability to clinical settings. This co-simulation framework could enable a detailed investigation of the physiological sources of seizures. Understanding the impact of the structure of the diseased area on seizure patterns and propagation would be of great interest (see e.g., Netoff et al., 2004; Garcia-Ramos et al., 2016). Specifically, we expect the inhibition and excitation ratios in the detailed neural network to play a critical role in seizure patterns (see e.g., Engel, 1996; Liu et al., 2020). Moreover, the Arbor-TVB user can implement various neural models and configuration topologies to simulate different brain regions and to computationally investigate diverse dynamic activities or the effects of medical interventions–for example, modeling subthalamic neurons along with synaptic and structural plasticity under stimulation in Parkinson's disease (Manos et al., 2021; Meier et al., 2022; Shaheen et al., 2022), Alzheimer's disease (Stefanovski et al., 2019; Manos et al., 2023) or tinnitus (Manos et al., 2018a,b) etc. Evidently, comparison, parameter tuning, and validation are also feasible using empirical neuroimaging data, however this was not the primary goal of this work. The simulated neural activity generated in each brain region of the connectome can be transformed into a BOLD signal-similar to the built-in functionality of TVB (Sanz Leon et al., 2013; Melozzi et al., 2017), which computes the hemodynamic response function (HRF) kernel (i.e., fMRI activity) using the Balloon-Windkessel model (Friston et al., 2000)—and can ultimately be aligned with neuroimaging time series data. Hence, a framework like Arbor-TVB can be extended to investigate various brain conditions.

## Data Availability

The codes necessary to reproduce selected results of this work are available on GitHub: https://github.com/arbor-contrib/arbor-tvb-cosim.
