# Supplementary material for: Arbor-TVB: a novel multi-scale co-simulation framework with a case study on neural-level seizure generation and whole-brain propagation
Source: Front Comput Neurosci. 2026 Feb 2;19:1731161. doi: 10.3389/fncom.2025.1731161 (PMC12907379; doi:10.3389/fncom.2025.1731161)
Supplement: Supplementary file 1 [file Data_Sheet_1.pdf]

Supplementary Material

1 RANDOM MORPHOLOGIES

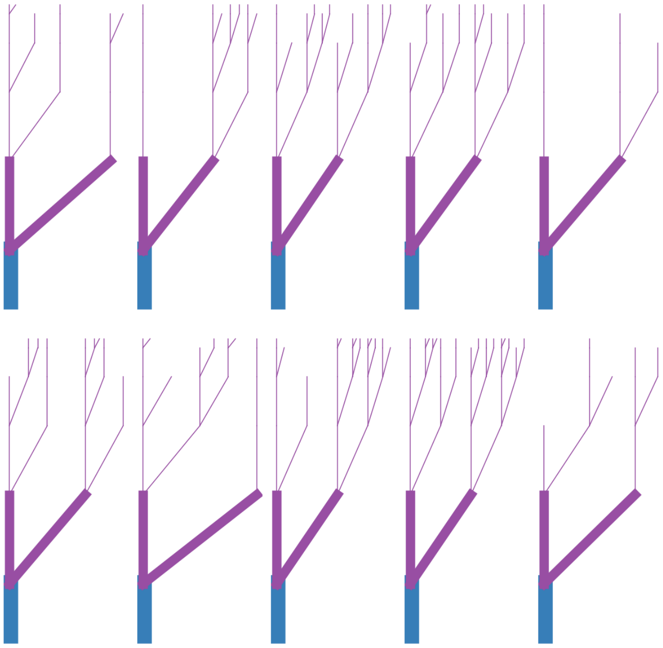

Figure S1. Dendrograms of the resultant morphologies generated using random seeds 0–10. Branch lengths are shown to scale; branch widths are proportional to their respective values.

2 RANDOM NETWORK REALIZATIONS

We confirmed that effects robust across network structure by using weights drawn from a normal distribution centered at $\mu = 0.5$ with width $\sigma = 0.5$, truncated to positive weights. The activity was averaged across 20 different realizations, see **Figure** S2 for the activity traces of the regions shown in **Figure** 8.

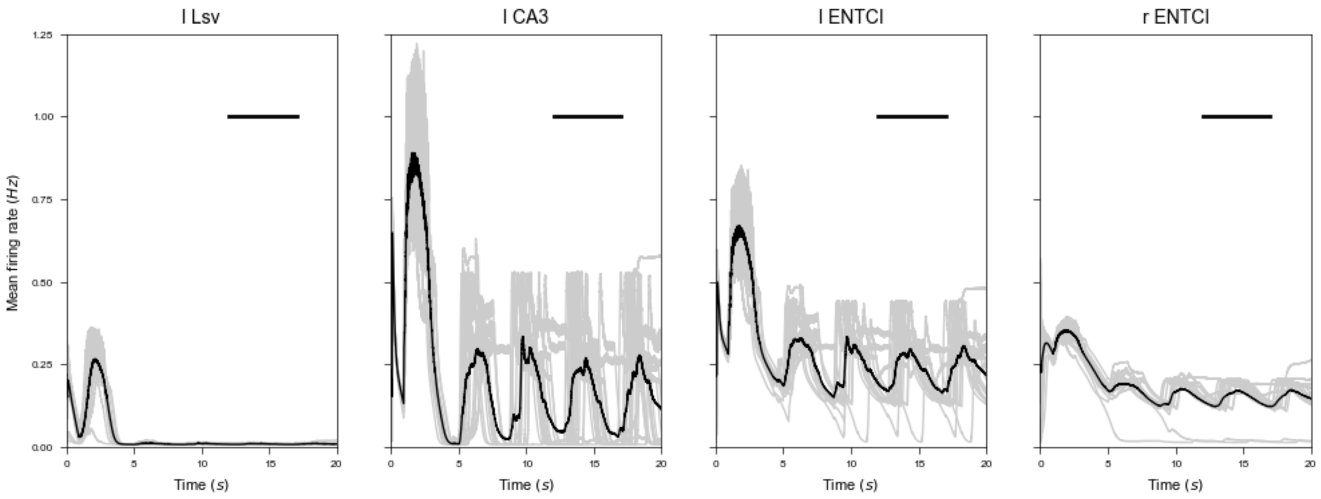

Figure S2. Individual traces (gray) of 20 randomly connected network realizations and their mean (black) over the full $20s$ simulation for the selected regions. A horizontal bar indicates the selected duration in the main text.

In a similar manner, the activity of the Arbor network was recorded and visualized, see **Figure** S3.

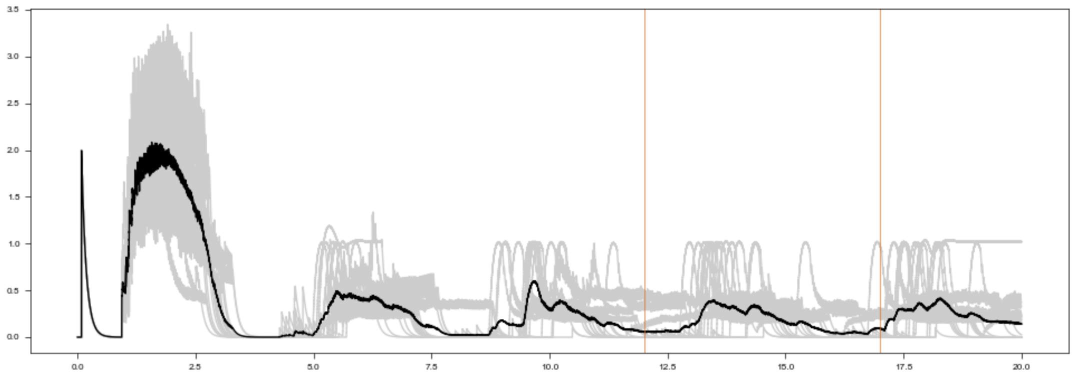

Figure S3. Individual traces (gray) of 20 randomly connected network realizations and their mean (black) over the full $20s$ simulation for the Arbor network. Vertical (orange) lines delineate the time slice shown in **Figure** 8 of the main text.
